# What can be done to reduce the burden of mental health problems globally?

**DOI:** 10.1017/gmh.2014.2

**Published:** 2014-09-11

**Authors:** Judith Bass

The ‘what can be done’ issue needs to be looked at from treatment and prevention angles.

On the treatment side, the availability of mental health professionals in low- and middle-income countries (LMIC) is quite limited and the availability of mental health services outside of urban settings and hospital settings is minimal (Kakuma *et al.* 2011; WHO Mental Health Atlas, 2011). This has resulted in a transition toward providing mental health services in non-specialized settings and ‘Task Sharing’ models for service provision, that is, services that may traditionally be provided by professionals with specialized mental health training are delegated to non-mental health workers. A recent Cochrane review of non-specialist health worker interventions for the care of mental, neurological and substance-abuse disorders in LMIC has shown that delivery of mental health services by non-mental health professionals in primary care and community-based settings can be an effective strategy for meeting the treatment gap (van Ginneken *et al.* 2013).

The World Health Organization (WHO) mhGAP program includes an intervention guide delineating a basic package of services for ten different categories of mental, neurological and substance use disorders (WHO, 2010): Depression, Psychosis, Epilepsy/Seizures, Developmental Disorders in Children/Adolescents, Behavioral Disorders in Children/Adolescents, Dementia in Older Adults, Alcohol Use Disorders, Drug Use Disorders, Self-Harm/Suicide, and the recently added Stress-Related Disorders (WHO, 2013). Although the programs and interventions included in the mhGAP Intervention Guide are informed by research, there is still a need for research to understand how these interventions can be made effective and accessible across LMIC settings to close the treatment gap. Some of the intervention questions that need to be addressed include what are the best settings to provide the different services, who is best positioned to implement the different services, how will the services be integrated into other health and social service systems (i.e. schools), what are the costs associated with the different services delivered in different settings, and how will the different services be paid for to ensure sustainability. There is also a need for research on how the implementation of these interventions may need to be adapted in contexts of instability and ongoing conflict.

The populations suffering from mental health problems in LMIC suffer the additional burden of poverty and the associated physical and social problems related to the diminished availability of resources (Lund *et al.* 2010). For that reason, it is important that future intervention research not only investigate the impact of mental health services on the remission of mental health symptoms, but also look at how interventions impact comorbid physical health problems, general functionality, social capital factors and outcomes related to economic development.

Although there is a growing research literature on the impact of intervention strategies, we are only at the beginning stages of understanding the impact of strategies to prevent mental health problems in LMIC. Thus, this is an area ripe for research and with the potential to improve the lives at the population level in LMIC settings. In exploring prevention strategies, it will be important to look beyond the health care field and explore the potential preventive impacts of education, social service, and economic programs as well as prevention strategies that specifically focus on ameliorating the risks related to mental health problems, such as violence prevention programs. The prevention field also has the opportunity to understand how interventions for one set of health conditions, such as HIV-prevention strategies, can have mental health impacts, for example through changing risky behaviors that increase risk for a range of mental and behavioral problems.

The ideas presented above represent just a beginning set of thoughts on where the field of global mental health prevention and intervention research is headed. This journal will seek to present research on emerging trends, innovative strategies and thoughtful reviews to help move our global knowledge forward on what works, and what does not work, to meet the mental health needs of LMIC populations.
